# Rare Gastric Diverticulum Mimicking Adrenal Abscess on Computed Tomography

**DOI:** 10.7759/cureus.55796

**Published:** 2024-03-08

**Authors:** Alexander Maraveyas

**Affiliations:** 1 Internal Medicine, Icahn School of Medicine at Mount Sinai Morningside-West, New York, USA

**Keywords:** ct, mimic, adrenal mass, adrenal pseudotumor, gastric diverticulum

## Abstract

Gastric diverticula are a rare phenomenon that is typically asymptomatic and encountered incidentally. Due to the relative proximity between the gastric diverticula and the left adrenal gland, they may mimic adrenal masses on computed tomography (CT). For this reason, the preferred diagnostic methods for gastric diverticula are upper gastrointestinal series or direct visualization on endoscopy. The present report describes an unusual case of a gastric diverticulum mimicking an abscess of the adrenal gland with the apparent spread of infection to the left lower lobe of the lung.

## Introduction

Gastric diverticula are exceptionally rare, with a reported prevalence of 0.02% at autopsy and 0.04% in the upper gastrointestinal series [1]. Due to the relative proximity of gastric diverticula to the left adrenal gland, they have been recognized as a rare mimic of masses of the left adrenal gland on computed tomography (CT) [2]. Consequently, the recommended diagnostic methods for gastric diverticula are upper gastrointestinal series or direct visualization on endoscopy [3,4]. This case is described as an unusual case of a gastric diverticulum mimicking an abscess of the adrenal gland with the apparent spread of infection to the left lower lobe of the lung.

## Case presentation

**Figure 1:**
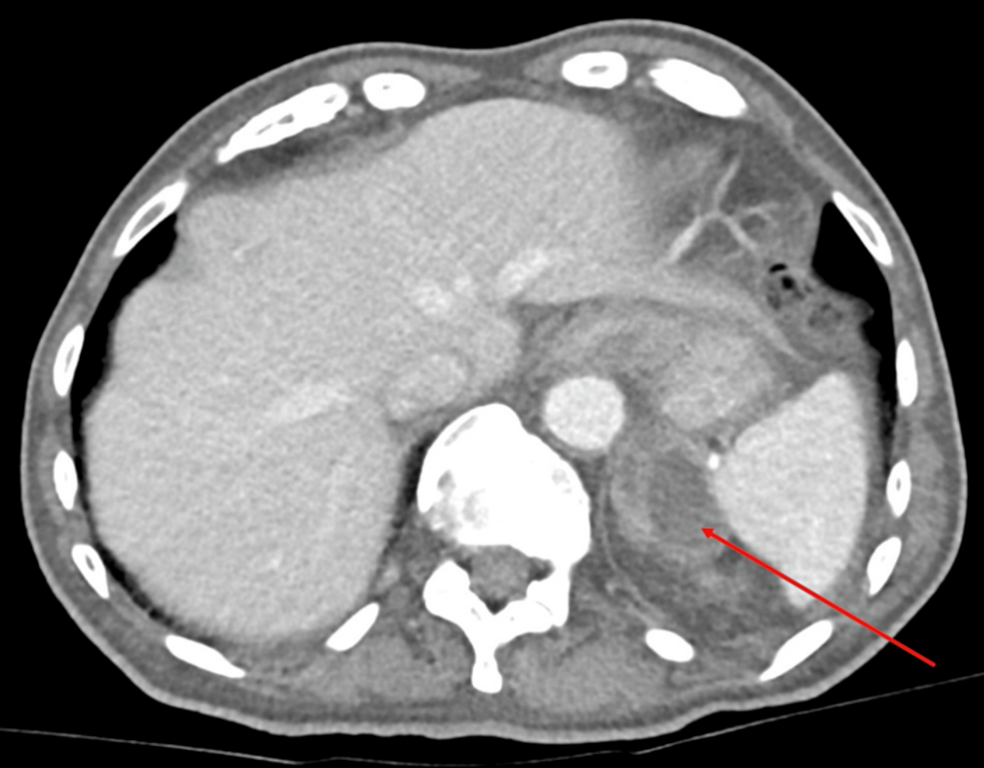
CT abdomen and pelvis with contrast Axial view demonstrating an apparent abscess arising from the left adrenal gland (red arrow); in reality, a gastric diverticulum arising from the posterior fundus of the stomach CT, computed tomography

A 78-year-old male with a history of colonic diverticulosis presented to the emergency department complaining of left flank discomfort and no other symptoms. Blood tests revealed mild anemia and leukopenia but were otherwise unremarkable; urinalysis was also unremarkable. A chest X-ray showed a left lower lobe opacity, possibly suggestive of pneumonia. Contrast-enhanced CT of the abdomen and pelvis demonstrated an apparent subdiaphragmatic abscess arising from the left adrenal gland (Figure 1), with the apparent spread of infection to the adjacent left lower lung.

**Figure 2:**
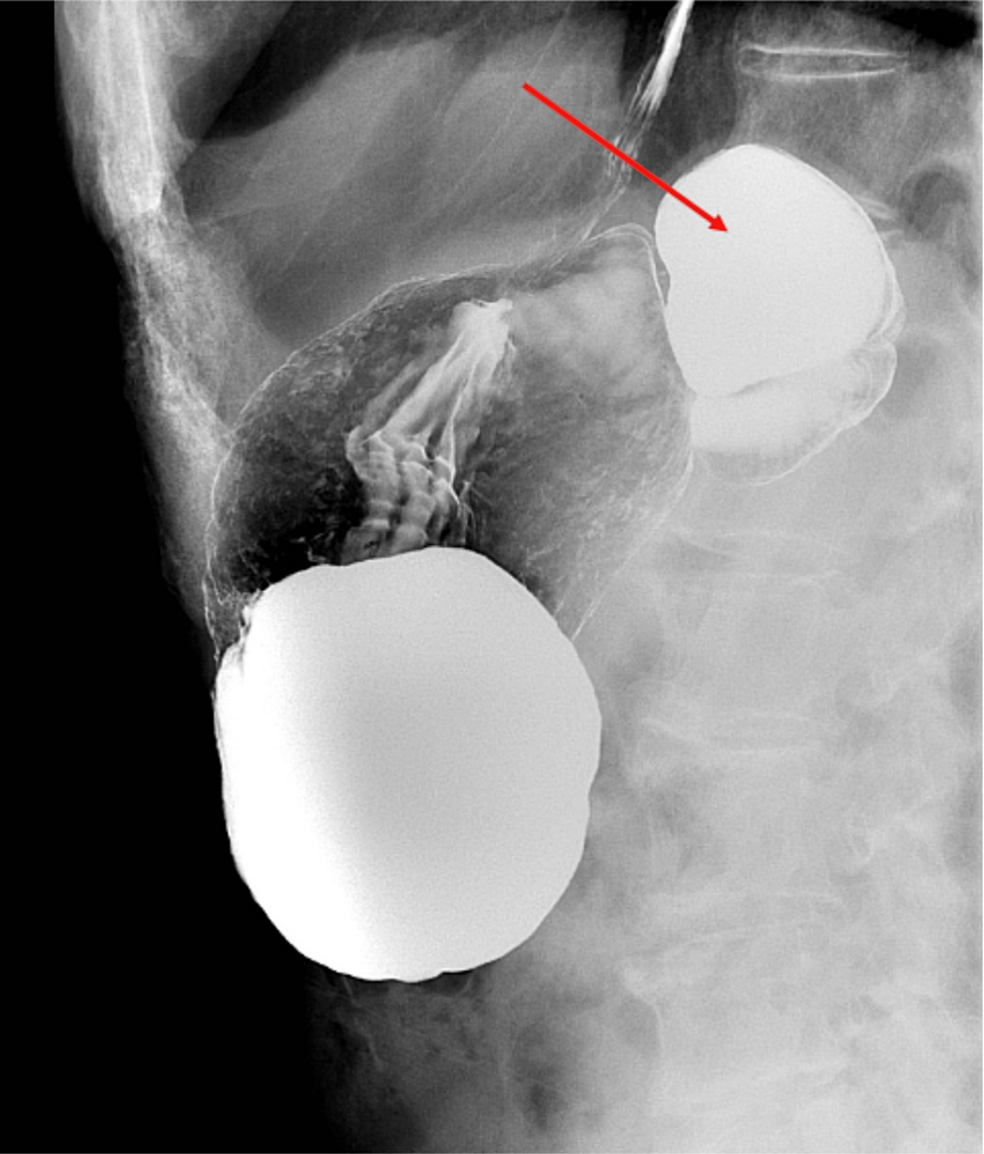
Upper gastrointestinal series Gastric diverticulum arising from the posterior fundus of the stomach (red arrow)

The patient was started on intravenous piperacillin-tazobactam. Interventional radiology and surgery services were consulted to evaluate the abscess. At this point, collateral information was received from a relative of the patient that this possible abscess had been present on scans performed 10 years ago at an outside hospital system; the family had been informed at the time that it was gastric in origin. On receipt of this information, the CT images were reevaluated by radiology, and gastric diverticulum was determined to be the most likely cause for both the appearance on the CT and the patient’s presentation. The patient, who had remained stable and afebrile throughout this admission, was discharged to follow-up outpatient gastroenterology. An upper gastrointestinal series was performed as an outpatient, which confirmed the diagnosis: gastric diverticulum arising from the posterior fundus of the stomach (Figure 2).

## Discussion

Gastric diverticula are a rare phenomenon, typically asymptomatic, and most commonly encountered incidentally. They are divided into two main types: congenital (or true) diverticula, which contain all layers of the abdominal wall, and acquired (or false) diverticula, which do not [5]. The congenital type is the more common, comprising an estimated 75% of cases [3]. The most common location for congenital diverticula is the posterior wall of the stomach, approximately 2 cm below the gastroesophageal junction and 3 cm from the lesser curve of the stomach [4].

Although gastric diverticula are a principally asymptomatic phenomenon, when symptomatic, the clinical picture is usually nonspecific. The most common symptom is the vague sensation of fullness or epigastric discomfort, although patients may also present with serious complications of gastric diverticula, including gastrointestinal hemorrhage [4]. In our case, the presenting symptom of left flank discomfort is broadly consistent with a typical symptomatic presentation.

The preferred techniques for diagnosing gastric diverticula are upper gastrointestinal series or direct visualization on endoscopy. When seen on CT, the typical appearance is a thin-walled cystic lesion located in the left paravertebral region [6], although, as this case illustrates, CT is not a preferred modality because of the potential for misdiagnosis. A 2015 review of the literature identified seven reported cases of gastric diverticula misdiagnosed as adrenal masses; of these, six had initially used CT to visualize the lesion [7]. Misdiagnosis can have deleterious effects on investigation and management and has been documented to have led to unnecessary endocrinological investigation [5], exploratory laparotomy [1], and even adrenalectomy [8].

The first-line treatment for gastric diverticula is medical management with either proton pump inhibitor therapy, H2 receptor antagonist therapy, or antacids [5]. Surgical resection is the treatment of choice when gastric diverticula are particularly large, symptomatic, or complicated by bleeding, perforation, or malignancy [9].

## Conclusions

This case demonstrates the importance of exercising caution when interpreting apparent adrenal masses as visualized on a CT scan. Additionally, the case highlights the importance of obtaining correlative studies, such as upper gastrointestinal series, when there is doubt regarding the provenance of an adrenal mass. We hope this case contributes to the literature on this uncommon phenomenon and raises the index of suspicion for gastric diverticula, in particular, as a cause of adrenal pseudotumor.
